# Supplementary material for: Deletion of wfs1 Impairs Oligodendrocyte Precursor Cells Dorsal Distribution and Myelination Through the wfs1-hmgcs1 Axis in Zebrafish
Source: Biology (Basel). 2026 Aug 16;15(16):1402. doi: 10.3390/biology15161402 (PMC13510151; doi:10.3390/biology15161402)
Supplement: Supplementary file 1 [file biology-15-01402-s001.zip › File S1-Transcriptome sequencing data and differentially expressed genes (DEGs) in wfs1-- zebrafish at 5 dpf/2.KEGG/wfs1KOvsWT/GSEA/WT/VIRAL_LIFE_CYCLE___HIV_1/VIRAL_LIFE_CYCLE___HIV_1(DRE03250).html]

Details for gene set VIRAL\_LIFE\_CYCLE\_\_\_HIV\_1(DRE03250)[GSEA]

|  || Dataset | deg.sample |
| Phenotype | sample.cls |
| Upregulated in class | WT |
| GeneSet | VIRAL\_LIFE\_CYCLE\_\_\_HIV\_1(DRE03250) |
| Enrichment Score (ES) | -0.25628963 |
| Normalized Enrichment Score (NES) | -1.1569065 |
| Nominal p-value | 0.22709164 |
| FDR q-value | 0.5224 |
| FWER p-Value | 1.0 |
Table: GSEA Results Summary

  

Fig 1: Enrichment plot: VIRAL\_LIFE\_CYCLE\_\_\_HIV\_1(DRE03250)      
 Profile of the Running ES Score & Positions of GeneSet Members on the Rank Ordered List

  

| PROBE | DESCRIPTION (from dataset) | GENE SYMBOL | GENE\_TITLE | RANK IN GENE LIST | RANK METRIC SCORE | RUNNING ES | CORE ENRICHMENT || 1 | 566841 | crebbpa |  |  | 530 | 2.904 | 0.0547 | No |
| 2 | 64275\_1 | supt5h |  |  | 705 | 2.577 | 0.1125 | No |
| 3 | 100147988 | chmp6a |  |  | 2217 | 1.281 | 0.0964 | No |
| 4 | 30731 | smarcb1b |  |  | 2781 | 1.086 | 0.1054 | No |
| 5 | 360142 | mxa |  |  | 3870 | 0.855 | 0.0922 | No |
| 6 | 799982\_1 | cd4-1 |  |  | 4912 | 0.692 | 0.0765 | No |
| 7 | 793232 | LOC793232 |  |  | 5184 | 0.657 | 0.0841 | No |
| 8 | 553453 | samhd1 |  |  | 5421 | 0.638 | 0.0923 | No |
| 9 | 337663 | mxe |  |  | 6254 | 0.562 | 0.0800 | No |
| 10 | 799716\_1 | smarcb1a |  |  | 6361 | 0.551 | 0.0901 | No |
| 11 | 559273\_1 | ep300a |  |  | 6707 | 0.519 | 0.0920 | No |
| 12 | 565533 | map1sb |  |  | 6861 | 0.504 | 0.0996 | No |
| 13 | 566591 | furinb |  |  | 7139 | 0.479 | 0.1026 | No |
| 14 | 114447 | cxcr4b |  |  | 7160 | 0.476 | 0.1137 | No |
| 15 | 550517 | mllt3 |  |  | 7443 | 0.457 | 0.1161 | No |
| 16 | 559032 | map1b |  |  | 8506 | 0.402 | 0.0926 | No |
| 17 | 415179 | tsg101a |  |  | 8716 | 0.387 | 0.0955 | No |
| 18 | 325221 | ell |  |  | 9139 | 0.357 | 0.0910 | No |
| 19 | 321464 | ccnt2b |  |  | 9154 | 0.356 | 0.0993 | No |
| 20 | 360143 | mxb |  |  | 9186 | 0.354 | 0.1070 | No |
| 21 | 797545 | map1ab |  |  | 9273 | 0.348 | 0.1129 | No |
| 22 | 100535683 | si:ch73-267c23.10 |  |  | 9410 | 0.339 | 0.1169 | No |
| 23 | 393137 | nelfb |  |  | 10386 | 0.280 | 0.0931 | No |
| 24 | 553760 | psip1a |  |  | 10626 | 0.265 | 0.0921 | No |
| 25 | 561067 | mxf |  |  | 10850 | 0.252 | 0.0913 | No |
| 26 | 565484 | mllt1a |  |  | 11104 | 0.239 | 0.0892 | No |
| 27 | 100004988 | si:ch211-207g17.2 |  |  | 11725 | 0.205 | 0.0748 | No |
| 28 | 140747 | cxcr4a |  |  | 13072 | 0.136 | 0.0358 | No |
| 29 | 394165 | nelfcd |  |  | 13163 | 0.132 | 0.0362 | No |
| 30 | 100005081 | si:ch211-207g17.3 |  |  | 13175 | 0.131 | 0.0391 | No |
| 31 | 322997 | ell2 |  |  | 13300 | 0.125 | 0.0383 | No |
| 32 | 567111 | crebbpb |  |  | 13470 | 0.117 | 0.0358 | No |
| 33 | 445397 | chmp6b |  |  | 14023 | 0.091 | 0.0207 | No |
| 34 | 327568 | ccnt2a |  |  | 14057 | 0.089 | 0.0219 | No |
| 35 | 565819 | LOC565819 |  |  | 14082 | 0.088 | 0.0233 | No |
| 36 | 558638 | zgc:162200 |  |  | 14565 | 0.067 | 0.0097 | No |
| 37 | 406705 | fez1 |  |  | 14690 | 0.061 | 0.0073 | No |
| 38 | 100330881 | zgc:171572 |  |  | 14802 | 0.055 | 0.0052 | No |
| 39 | 558395 | xpo1a |  |  | 15460 | 0.024 | -0.0148 | No |
| 40 | 565612 | ep300b |  |  | 15569 | 0.019 | -0.0178 | No |
| 41 | 100330316 | LOC100330316 |  |  | 15933 | 0.001 | -0.0292 | No |
| 42 | 406835 | serinc5 |  |  | 16038 | -0.003 | -0.0324 | No |
| 43 | 559273 | ep300a |  |  | 16774 | -0.025 | -0.0549 | No |
| 44 | 799716 | smarcb1a |  |  | 16998 | -0.034 | -0.0610 | No |
| 45 | 393880 | vps4b |  |  | 17091 | -0.036 | -0.0631 | No |
| 46 | 556758 | bicd1a |  |  | 17807 | -0.068 | -0.0839 | No |
| 47 | 571621 | nup153 |  |  | 17848 | -0.069 | -0.0834 | No |
| 48 | 767670 | vps4a |  |  | 17948 | -0.073 | -0.0847 | No |
| 49 | 100317326 | si:ch1073-55a19.2 |  |  | 17968 | -0.074 | -0.0835 | No |
| 50 | 566557\_1 | furina |  |  | 18036 | -0.078 | -0.0837 | No |
| 51 | 327069 | cpsf6 |  |  | 18096 | -0.081 | -0.0836 | No |
| 52 | 556421 | aff4 |  |  | 18268 | -0.088 | -0.0868 | No |
| 53 | 406669 | pdcd6ip |  |  | 18443 | -0.096 | -0.0899 | No |
| 54 | 561138 | xpo1b |  |  | 18716 | -0.109 | -0.0958 | No |
| 55 | 559273\_2 | ep300a |  |  | 19072 | -0.125 | -0.1039 | No |
| 56 | 393164 | chmp4ba |  |  | 19448 | -0.142 | -0.1122 | No |
| 57 | 30572 | ran |  |  | 20453 | -0.185 | -0.1392 | No |
| 58 | 100333959 | si:ch211-233g6.2 |  |  | 20467 | -0.186 | -0.1351 | No |
| 59 | 394062 | tnpo3 |  |  | 20656 | -0.196 | -0.1362 | No |
| 60 | 100333057 | tsg101b |  |  | 20662 | -0.197 | -0.1315 | No |
| 61 | 567111\_1 | crebbpb |  |  | 20683 | -0.198 | -0.1273 | No |
| 62 | 100004625 | ccr11.1 |  |  | 21302 | -0.230 | -0.1410 | No |
| 63 | 334465 | ccnt1 |  |  | 22054 | -0.270 | -0.1580 | No |
| 64 | 335519 | ppiab |  |  | 22648 | -0.305 | -0.1692 | No |
| 65 | 563942 | kat2b |  |  | 23122 | -0.336 | -0.1758 | No |
| 66 | 678639 | mllt1b |  |  | 23583 | -0.365 | -0.1813 | No |
| 67 | 436782 | supt4h1 |  |  | 25028 | -0.448 | -0.2157 | No |
| 68 | 555517 | kat2a |  |  | 25644 | -0.498 | -0.2228 | No |
| 69 | 436648 | nelfe |  |  | 25802 | -0.511 | -0.2152 | No |
| 70 | 559677 | nelfa |  |  | 26176 | -0.546 | -0.2135 | No |
| 71 | 562203 | si:dkeyp-110c12.3 |  |  | 27223 | -0.645 | -0.2305 | No |
| 72 | 557383 | bicd2 |  |  | 27963 | -0.737 | -0.2357 | Yes |
| 73 | 100151220 | map1aa |  |  | 28620 | -0.842 | -0.2356 | Yes |
| 74 | 559032\_1 | map1b |  |  | 29070 | -0.936 | -0.2268 | Yes |
| 75 | 566557 | furina |  |  | 29268 | -0.994 | -0.2085 | Yes |
| 76 | 562808\_1 | map1sa |  |  | 29297 | -0.999 | -0.1849 | Yes |
| 77 | 64275 | supt5h |  |  | 29330 | -1.009 | -0.1611 | Yes |
| 78 | 393721 | pin1 |  |  | 29925 | -1.214 | -0.1500 | Yes |
| 79 | 555772 | ftr52p |  |  | 30221 | -1.350 | -0.1261 | Yes |
| 80 | 559677\_1 | nelfa |  |  | 30425 | -1.462 | -0.0966 | Yes |
| 81 | 566841\_1 | crebbpa |  |  | 30571 | -1.563 | -0.0628 | Yes |
| 82 | 334175 | hgs |  |  | 30624 | -1.608 | -0.0249 | Yes |
| 83 | 321602\_1 | cdk9 |  |  | 31394 | -2.633 | 0.0156 | Yes |
Table: GSEA details [plain text format]

  

Fig 2: VIRAL\_LIFE\_CYCLE\_\_\_HIV\_1(DRE03250)      
 Blue-Pink O' Gram in the Space of the Analyzed GeneSet

  

Fig 3: VIRAL\_LIFE\_CYCLE\_\_\_HIV\_1(DRE03250): Random ES distribution      
 Gene set null distribution of ES for **VIRAL\_LIFE\_CYCLE\_\_\_HIV\_1(DRE03250)**
